# An evaluation of the Gemsaec 3E centrifugal analyser

**DOI:** 10.1155/S1463924681000254

**Published:** 1981

**Authors:** Peter S. West, Jennifer A. Nisbet, John A. Owen

**Affiliations:** Department of Chemical Pathology St. George's Hospital London SW17 UK


reprocess the data and skip drop-outs. A non-linear least square fit was required to calculate the calibration function. With quantitative GC-MS methods curved calibration lines not passing through the origin are often encountered [7, 8]. If the calibration is considered to be correct, the computer lists the remaining data blocks while the user has to supply information on the amount of internal standard added and the volume of plasma sample initially processed. After the sample identification, the system presents the final report, a typical example of which is shown in Figure 6.

### Conclusions

A flexible system for routine quantitative GC-MS analyses was developed using the Varian SpectroSystem 100MS. The software was extended with user-oriented BASIC programs. With the BASIC programs loaded from magnetic tape, access to integrated data stored on disc was obtained. The system provides an interrogation dialogue for the user to set criteria for data selection, to calculate a calibration function and to calculate the final result of analysis. The system fulfilled the demands on quality control on the results of analyses for the quantitative determination of low drug levels in biological samples.

*Note added in proof*

*The GC-MS system as described in this paper was recently extended with a Hewlett-Packard 7670 A automatic sampler for injection of samples into the GC column. The autosampler was activated by a homemade acoustic pulse counter. The acoustic signals were generated by the Tektronix 4010 terminal under control of the Varian DISKOS software using the module BELL.*

ACKNOWLEDGEMENTS

The authors wish to thank Mr. J. Eberhard (Ingenieursbüro für Datentechnik Dr. Ing. H. Seufert, Karlsruhe, G. F. R.) and Mr. U. Markwardt and Mr. M. Schmädecke (Varian MAT GmbH, Bremen, G. F. R.) for their information on the organisation of the DISKOS software.

REFERENCES

[1] De Leenheer, A. P. and Roncucci, R. R., (1977), "Quantitative Mass Spectrometry in Life Sciences", Elsevier, Amsterdam.

[2] De Leenheer, A.P., Roncucci, R. R. and Van Peteghem, C., (1978), "Quantitative Mass Spectrometry in Life Sciences II", Elsevier, Amsterdam.

[3] Millard, B. J., (1978), "Quantitative Mass Spectrometry", Heyden, London.

[4] Carrington, R. and Frigerio, A., (1977), *Drug Metabolism Reviews*, **6**, 243.

[5] Beynon, J. H., (1978), *Pure and Applied Chemistry*, **50**, 65.

[6] Vink, J. and Van Hal, H. J. M., in "Quantitative Mass Spectrometry in Life Sciences II", Ed. De Leenheer, A. P., Roncucci, R. R. and Van Peteghem, C., 1978, Elsevier, Amsterdam, pp. 367–378.

[7] Picart, D., Jacolot, F., Berthou, F. and Floch, H. H., *ibid*, pp. 105–118.

[8] Pickup, J. F. and McPherson, K., (1976), *Analytical Chemistry*, **48**, 1885.

### An evaluation of the Gemsaec 3E centrifugal analyser

**Peter S. West, Jennifer A. Nisbet and John A. Owen**

*Department of Chemical Pathology, St. George's Hospital, London SW17, UK.*

### Introduction

Centrifugal analysers have been progressively developed since they were first introduced. The Gemsaec 3E centrifugal analyser (Electro-Nucleonics International Ltd., Breda, Holland) has a number of innovations. In place of filters, a grating monochromator provides monochromatic light over a wide range and a solid state photodiode allows measurement of light absorption with low electronic noise. There is improved temperature control within the rotor and the rotor wash cycle uses water only, which avoids the toxic and fire hazard of methanol used in other models. Finally, it has its own computer with a floppy disc mass storage providing a wide range of programs including a statistical package and provision for storage and recall of a large amount of analytical data.

The analytical performance of the Gemsaec 3E centrifugal analyser has been assessed and the results are presented here together with comparative published data relating to similar instruments.

### Description of the instrument

The Gemsaec 3E centrifugal analyser comprises five modules - rotoloaders, analyser, control module, computer and keyboard printer. It uses 16-place re-usable transfer discs.

The rotoloader comprises two automatic pipetting units (MicroMedic Systems, Inc. Horsham, U.S.A.) one for dispensing reagent (200–600μl) and the other for diluting sample (3–50μl) with diluent (20–200μl). Loading a transfer disc takes about 3 minutes.

The analyser has an optical system consisting of a diffraction grating monochromator with a wavelength range from 335 to 785 nm and a band width of 5 nm. The temperature of the rotor is adjustable between 20 and 40°C.

The control module allows selection of the various test parameters including temperature, time of first absorbance reading and intervals between readings. It contains an oscilloscope display which allows visual monitoring of reactions in progress.

The computer (LS11, Digital Equipment Corporation London) has 40K bytes of memory and a twin disc drive unit as mass store. The keyboard printer (LA36 Digital Equipment Corporation London) has a rating of 30 char/s and is used to operate the system and to display and manipulate results.

### Evaluation procedure

### Analytical methods

The tests used in evaluating the instrument were selected so as to involve a number of different analytical principles, viz.

albumin by dye binding, albumin by immunochemical precipitation, aspartate aminotransferase (AST), creatine kinase, creatinine and triglyceride by kinetic methods. Total protein was measured by conventional colorimetry and bilirubin by bichromatic spectrophotometry which involved taking absorbance readings at 540 nm, changing the wavelength manually without stopping the rotor and then taking absorbance readings at 454 nm. Difference in absorbance readings for each sample position were calculated automatically. The procedures used were as described in the instruction sheets supplied by the manufacturers except as follows :

(a) albumin (dye-binding): bromocresol green reagent (Technicon Instrument Company Ltd., Basingstoke, U.K.) was used.
(b) albumin (immunochemical): for convenience, samples were diluted 1:200 (50$\mu$l sample + 10ml sodium chloride 9g/1).
(c) aspartate aminotransferase: Boehringer reagents (kit No. 191337) were used. Tris buffer-substrate was dissolved in 87ml water and $\alpha$-oxoglutarate in 10ml water. Before use, 8.7ml buffer substrate was mixed with 0.3ml $\alpha$-oxoglutarate solution. Two alternative procedures were used in calculating AST activities. Each involved 4 absorbance readings at 30s intervals. One (as recommended for use with phosphate buffer) took the first reading at 90s after mixing, the second at 240s.
(d) creatinine: after addition of picric acid to the sodium hydroxide, the mixture was left for 10 min before the addition of distilled water. The final mixture was left for 30 min before use.
(e) creatine kinase: Boehringer reagents (kit No.181188), with N-acetylcysteine as activator, were used.
(f) total protein: Boehringer reagents (kit No.124381) were used. A blank value was obtained for every sample using sodium hydroxide-tartrate in place of biuret reagent.
(g) triglyceride: Boehringer reagents (kit No.24473) were used with ATP tablets (No.193585). A reagent blank was determined and subtracted from standard and test readings.

The test procedures, apart from the enzyme assays, were calibrated with one standard except in the case of immunochemical albumin for which 4 standards were used since calibration was non-linear throughout. The enzyme tests were calibrated in terms of absorbance change. Copies of the method instruction sheets supplied by the manufacturer are available from us on request.

### Precision

For each test, precision was measured at low, medium and high assay levels taking 4 samples at each level (3 in the case of immunochemical albumin) on each of 20 days.

The assay materials were commercial lyophilised serum (Table 1) which were reconstituted with distilled water at the start of the evaluation period. The contents of bottles were pooled and then divided into portions of 2ml per test per day. These sera were stored at $-20^\circ$C until the day of analysis when they were thawed, mixed and kept at $4^\circ$C until required. The serum for creatine kinase at the medium assay level was reconstituted fresh each day.

Precision data were expressed as the coefficient of variation. Results lying more than 3 standard deviations from the mean on initial analysis of data, were excluded from the final calculations.

### Accuracy

This was assessed by comparing Gemsaec results for patient specimens with those obtained on routine analytical procedures (Table 2) calibrated, except in the case of bilirubin, with the same standards or on the same basis (enzymes tests) as in the Gemsaec procedure.

**Table 1. Assay materials used in precision studies**
Results are mean values obtained in this study. Code letters indicate nature of materials as follows: A Lipidtrol, B Monitrol IE, C Monitrol IIE, D Precilip, E Precipath, E F Validate, G Validate A, H Versatol Hi, I Wellcomtrol I, J Wellcomtrol III.

| test | units | standard values | assay level low | medium | high |
|---|---|---|---|---|---|
| albumin (dye binding) | g/l | 42 | 25 G | 29 I | 39 F |
| albumin (immuno-chemical) | g/l | 14,28, 42,70 | 23 G | 31 F | 40 B |
| AST | IU/1 | – | 15 F | 78 I | 137 E |
| bilirubin | $\mu$mol/1 | 307 | 70 I | 132 H | 391 J |
| creatinine | $\mu$mol/1 | 200 | 71 F | 227 G | 484 H |
| creatine kinase | IU/1 | – | 29 F | 130 E | 311 C |
| total protein | g/l | 76 | 49 G | 63 J | 78 I |
| triglyceride | mmol/1 | 2.0 | 0.38 I | 1.70 D | 2.98 A |

**Table 2. Methods used in routine analysis**

| Test | Equipment | Reagents |
|---|---|---|
| albumin (dye binding) | AutoAnalyser II | Bromocresol Green (Technicon) |
| AST | LKB 8600 37°C | Boehringer kit No. 191331 |
| bilirubin | AutoAnalyser II | Caffeine, Diazo reagent Tartrate buffer |
| creatinine | AutoAnalyser II | Sodium Hydroxide Picric acid |
| creatine kinase | LKB 8600 37°C | Boehringer kit No. 126357 |
| total protein ø | Centrifichem | BDH Biuret Reagent Lot No. 20094 |
| triglyceride | LKB 8600 37°C | Boehringer kit No. 150606 |

*ø performed in another laboratory.*

### Calibration linearity

For each test, a lyophilised serum with an appropriately high value was reconstituted and 10 dilutions in equal steps made in sodium chloride (9g/1). The dilutions were analysed on the day of preparation or stored at $-20^\circ$C until the following day. In a few instances, linearity checks were also carried out using dilutions from patients samples or from aqueous standard solutions.

### Carryover

This was determined using the procedure of Young & Gochman [1]. Three successive identical samples of reconstituted serum with high value (H1, H2, H3) were loaded followed by three successive identical samples with low values (L1, L2, L3). The carryover (%) was obtained from the expression

$$\frac{L1 - L3}{H3 - L3} \times 100.$$

The splashover from one cuvette to another, as a component of carryover [2], was examined by placing 0.5ml of water of alkaline creatinine picrate solution in alternate positions of the transfer disc and running the disc for 60s after mixing. Splashover was expressed as the mean absorbance of the 'water' cuvettes as a percentage of the mean absorbance of the 'picrate' cuvettes.

### Results

### Precision

Data on analytical precision are given in Table 3. The coefficients of variation obtained over the whole evaluation period were less than 5% except at low assay levels of AST, creatinine and triglyceride. Total protein gave the most precise results. For all tests, except low level total protein, the precision within a day was better than over 20 days.

### Accuracy

A comparison of Gemsaec results and those obtained on the same patient specimens by routine laboratory methods is presented in Table 4. In general, there was good agreement between the two sets of results with very high correlation coefficients. AST results calculated from readings starting at 240s agreed better with results obtained by the routine method than those calculated from readings starting at 90s. To test whether this was due to the presence of endogenous substrate, activities were measured in 25 patient samples without added $\alpha$-oxoglutarate using readings starting at 90s. A mean activity of 15 (range 9–36) IU/l was obtained: negligible activity was obtained on analysing reconstituted sera without added $\alpha$-oxoglutarate.

### Linearity

The limits of linearity obtained on the Gemsaec with different materials are listed in Table 5. In some instances higher limits of linearity were obtained with pooled patient material or aqueous standards than with reconstituted sera.

### Carryover

Carryover with reconstituted sera was less than 1% for all tests except immunochemical albumin and creatinine (Table 6). The lowest values for carryover were with albumin (dye-binding) and bilirubin, two tests with a low sample to diluent ratio i.e. 3:100 and 15:100 respectively. The highest values occurred with creatinine. To try and explain this, carryover was also calculated for creatinine using patients samples or aqueous standards. This gave mean carryover values of 1.2% (1.1-1.3, n=2) and 0.7% (0.7-0.7, n=2) respectively.

To see if it contributed significantly to the high carryover with creatinine, splashover was measured with creatinine only and gave a mean value of 0.22% (0.16-0. 34,n=7).

### Discussion

Comparative analytical precision data published for various centrifugal analysers is presented in Table 7 which indicates that for most tests at most levels the precision which we obtained with the Gemsaec 3E was as good or better than that obtained by others. The Gemsaec 3E appears from data in Table 7 to be more precise in measuring bilirubin but this may not be a conclusion relevant to every day work, because the data of others was obtained using diazo methods whereas we used a direct spectrophotometric method.

The level of clinically desirable precision has been discussed by a number of authors [13,14,15,21]. Included in Table 7 are estimates based on their suggestions, which indicate that centrifugal analysers in general, and the Gemsaec 3E in particular, provide a generally acceptable level of analytical performance.

There was good agreement between the Gemsaec results for patients samples and results obtained by routine analytical procedures with AST. There was greater discrepancy between routine results and Gemsaec results obtained from readings starting at 90s than between routine results and Gemsaec results starting at 240s. It is possible that the higher Gemsaec values obtained from earlier readings were due to endogenous substrate which was used up as the reaction proceeded; certainly, an appreciable reaction was observed in the absence of added substrate. The routine method involved a preincubation period of approximately 15 min which would allow endogenous substrate to be used up before a definitive reaction was started. For measuring AST on a centrifugal analyser Ertingshausen et al [7] modified the pipettor unit to include a 10 minute preincubation.

Bilirubin results obtained by direct spectrophotometry on the Gemsaec agreed well with those obtained by a diazo reaction method run on an AutoAnalyzer. This is a little surprising considering that the direct spectrophotometric method is liable to interference from other pigments. It should be noted, however, that the calibrating standards for bilirubin used on the Gemsaec were not identical with the standards used to calibrate the routine method and, while

**Table 3. Precision data**
Results are expressed as coefficients of variation

| test | within day ø | | | overall (20 days) øø | | |
|---|---|---|---|---|---|---|
| | low | medium | high | low | medium | high |
| albumin (dye-binding) | 1.2 | 1.0 | 1.6 | 2.4 (1) | 1.5 | 1.8 |
| albumin (immuno-chemical) | 2.4 | 2.8 | 3.3 | 4.4 (1) | 4.3 | 3.8 (2) |
| AST | 8.0 | 2.4 | 1.6 | 9.4 (1) | 4.9 | 2.5 |
| bilirubin | 1.1 | 0.6 | 0.6 | 1.9 | 1.8 | 1.6 |
| creatinine | 4.9 | 1.8 | 1.8 | 6.1 | 3.0 (3) | 3.6 |
| creatine kinase | 3.0 | 3.2 | 2.5 | 4.7 (1) | 4.0 | 3.8 |
| total protein | 0.8 | 0.9 | 0.7 | 0.7 | 0.9 (2) | 1.0 |
| triglyceride | 5.0 | 1.9 | 1.1 | 6.6 (2) | 2.7 | 2.4 |

*ø based on 20 assays except high level creatine kinase which was based on 16.*

*øø based on 76-80 assays in each category except for immunochemical albumin which was based on 58-60 assays. Number of outliers in brackets.*

**Table 4. Comparison of patient results obtained on the Gemsaec with those by routine laboratory methods**
Patient results (routine) are expressed as mean, range and number. Slope (a) and intercept (b) refer to the line y = ax + b, which was calculated to express best the linear relation between Gemsaec value (y) and routine value (x).

| Test | patient results | Correlation Coefficient | Slope | Intercept |
|---|---|---|---|---|
| albumin (dye binding) | 34.2 13-46 n = 100 | 0.99 | 0.88 | + 1.7 |
| AST taking readings from a) 90s | 52.1 9-310 n = 96 | 0.99 | 0.98 | + 12.4 |
| taking readings from b) 240s | | 0.99 | 0.99 | + 4.9 |
| bilirubin | 66 6-576 n = 90 | 0.99 | 0.98 | + 0.9 |
| creatinine | 140 35-1684 n = 100 | 0.99 | 1.00 | - 3.4 |
| creatine kinase | 191 9-1177 n = 100 | 0.99 | 0.95 | + 6.3 |
| triglyceride | 1.7 0.6-8.5 n = 100 | 0.99 | 0.94 | - 0.03 |
| total protein | 66.8 46-87 n = 92 | 0.99 | 0.87 | + 5.9 |

the standards were believed to be consistent, there could have been a small discrepancy nullifying the average effect of non-bilirubin pigment in the Gemsaec procedure.

The creatinine results obtained on the Gemsaec were slightly lower than those obtained on the AutoAnalyzer possibly because the method run on the Gemsaec was kinetic and, as such, essentially specific [16, 17]. It has been observed, however, that measurement of creatinine on an AutoAnalyzer provides results much nearer the true creatinine value than measuring total picrate chromogen [18] and this may have accounted for the relatively small difference between the creatinine results obtained by the two methods.

Measuring albumin on the basis of dye binding gave a slightly lower mean patient value on Gemsaec than by the routine procedure. This may have been due, in part, to non-linearity of the albumin procedure at high assay values. However, exclusion of albumin results above the linearity limit did not abolish the difference. Another factor which may have been responsible for the difference was the reaction time (90 s) being shorter in the Gemsaec procedure than in routine procedure (200 s), since it has been shown [19] that plasma proteins other than albumin with dye binding properties, react with dyes more slowly.

Total protein results were generally lower on the Gemsaec but this may have been due to subtraction of a serum blank which was not done in the routine method. The mean blank for the patients samples was 1.5 (1-5) g/l which would largely account for the difference noted.

The slightly lower triglyceride values obtained on the Gemsaec may have been due to incomplete saponification of triglyceride during the pre-incubation period which was 10 min at room temperature in the Gemsaec procedure and longer at a higher temperature in the routine method.

The limits of calibration linearity are important when a procedure is calibrated by a single standard. We found that limits of linearity (Table 5) on the Gemsaec were generally acceptable though, for some tests, not as high as specified by the manufacturer. In some instances e.g. determination of albumin, this may have been due to our using a reagent other than that recommended but non-linearity in determining albumin on a centrifugal analyser has been noted previously [20]. Calibration linearity extended over a wider range in the routine method in which the same bromocresol green reagent was used. This may have been due to the greater dilution of plasma in the routine procedure (1:440) than in the Gemsaec procedure (1:170). Some tests (Table 5) gave higher limits of linearity with aqueous standards or with pooled patient plasma than they gave with reconstituted lyophilised serum. Unfortunately time during which the Gemsaec was available for our assessment was not long enough to allow us to investigate the various anomalies noted with the linearity studies.

For most tests carryover was small but it was appreciable in the case of creatinine. Splashover between cuvettes was thought to be a significant factor in producing the high value for creatinine carryover; however direct measurement of splashover indicated that it had a negligible contribution. No explanation is offered as to why carryover should be so much higher with creatinine than with other tests.

It is concluded that the precision and accuracy of the Gemsaec 3E centrifugal analyser are quite adequate for routine clinical chemistry. Admittedly, this assessment was based on only 8 different tests but the tests selected were based on various analytical principles and the authors have no reason to believe that the analytical performance of the instrument would be very different with other tests involving

**Table 5. Calibration linearity**

| test | materialø | unit | limit of linearity found in this study | limit of linearity specified by manufacturer |
|---|---|---|---|---|
| albumin (dye binding) | RS | g/l | 35 | 80* |
| AST | RS<br>PPP | IU/L | >145øø<br>340 | not stated |
| bilirubin | RS | μmol/l | 450 | not stated |
| creatinine | RS<br>AS | μmol/l | 320<br>2000 | 1800 |
| creatine kinase | RS<br>PPP | IU/l | 370<br>1000 | 1000 |
| total protein | RS | g/l | 100 | 120 |
| triglyceride | RS | mmol/l | >3.7øø | 5.7 |

*ø RS, reconstituted sera; PPP, pooled patients' plasma; AS, aqueous standard.*
*øø highest value attainable with reconstituted serum.*
** using 'Albustrate' bromocresol green reagent.*

**Table 6. Carryover data**

| test | No. | carryover (%) mean | carryover (%) range |
|---|---|---|---|
| albumin (dye binding) | 4 | 0 | – |
| albumin (immunochemical) | 2 | 1.60 | 0 – 3.2 |
| AST | 10 | 0.85 | 0 – 2.5 |
| bilirubin | 14 | 0.15 | 0 – 0.3 |
| creatinine | 16 | 3.00 | 1.4 – 4.4 |
| creatine kinase | 9 | 0.75 | 0 – 1.1 |
| total protein | 4 | 0.70 | 0 – 1.4 |
| triglyceride | 10 | 0.65 | 0 – 2.2 |

These data were obtained with reconstituted sera.

**Table 7. Comparative precision data for centrifugal analysers**
Data are expressed as the range of day to day coefficients of variations for different levels. References in parenthesis.

| | albumin (dye binding) | albumin (immunochemical) | AST | bilirubin | creatine kinase | creatinine | triglyceride | total protein |
|---|---|---|---|---|---|---|---|---|
| This study | 1.6–2.4 | 3.8–4.4 | 2.5–9.4 | 1.6– 1.9 | 3.8–4.7 | 3.0–6.1 | 2.4–6.6 | 0.7–1.0 |
| Published data | 2.0–2.4(3) | 2.4–4.0(5) | 2.0–8.4(3) | 7.6–24.5(3) | | 4.3(10) | 2.1–4.4(12) | 0.9–1.4(3) |
| | 2.5–3.4(4) | | 1.4–3.8(6) | 2.4– 3.2(6) | | 2.0–5.9(11) | | 1.7–1.9(4) |
| | | | 2.8–5.2(7) | | | | | |
| | | | | 3.2– 3.8(8) | | | | |
| | | | | 3.3(9) | | | | |
| Clinically desirable | 4 | 4 | 10 | – | – | – | – | 3(13)ø |
| | 6 | 6 | – | – | – | – | – | 7(14)øø |
| | 6 | 6 | 4 | 3 | – | 4 | – | 2(15)øøø |
| | 7 | 7 | 8 | – | – | – | – | 4(21)øøø |

basis of estimate *ø individual variation; øø normal range; øøø subjective view.*

the same principles. The instrument is easy to use once operators become familiar with its operation. Although the instrument was then evaluated for a few weeks no faults occurred which would raise doubts about its reliability in everyday use.

ACKNOWLEDGEMENTS
We thank the American Hospital Supply (U.K.) Ltd., for the loan of the instrument and the Department of Health for the financial support to enable us to carry out this evaluation. We are also grateful to Mr. J. Korten (Electronucleonics, Breda, Holland) and Mr. I. West (American Hospital Supply Company) for advice and assistance throughout the evaluation and to Mr. P. Brooks (Mayday Hospital, Croydon) for providing patient samples assayed for total protein.

REFERENCES
[1] Young, D. S. and Gochman, N. Standard methods of Clinical Chemistry, 1972, 7, 293.
[2] Henry, P., *Journal of Automatic Chemistry*, (1979), **1**, 195.
[3] Henry, P. and Saunders, R.A. *Annals of Clincial Biochemistry*, 1976, 13, 384.
[4] Savory, J., Heintges, M. G., Sonowane, M., and Cross, R. E. *Clinical Chemistry*, (1976), **22**, 1102.
[5] Blom, M. and Hjorne, N. *Clinical Chemistry*,(1975), **21**,195.
[6] Fleetwood, J. A., Latner, A. L., Marriner, A., Skillen, A. W. and Smith, P. A. *Annals of Clinical Biochemistry*, (1977), **14**, 279.
[7] Ertingshausen, G., Amsellem-Winzelberg, L., Richert, J-F., and Davids, R. *Clinical Chemistry*, (1978), **24**, 1147.
[8] Brody, J. P., Valdes, R., Jnr. and Savory, J. *Clinical Chemistry*, (1979), **25**, 205.
[9] Cross, R. E., Heintges, M. G., Savory, J. and Wentz, P. W. *Clinical Chemistry*, (1976), **22**, 429.
[10] Van Stekelenburg, G. J., Valk, C., Van De Kamp, J. S., Van Wijngaarden-Penterman, M. J. G., and De Keijzer, M. H. *Clinica Chimica Acta*, (1978), **89**, 79.
[11] Fabiny, D. L., Ertingshausen, G., *Clinical Chemistry*, (1971), **17**, 696.
[12] Wentz, P. W., Cross, R. E. and Savory, J. *Clinical Chemistry*, (1976), **22**, 188.
[13] Cotlove, E., Harris, E. K. and Williams, G. Z. *Clinical Chemistry*, (1970), **16**, 1028.
[14] Tonks, D. B. *Clinical Chemistry*, (1963), 9, 217.
[15] Campbell, D. G. and Owen, J. A. *Australasian Annals of Medicine*, (1969), **18**, 4.
[16] Larsen, K. *Clinica Chimica Acta*, (1972), **41**, 209.
[17] Knoll, Von E., Rebel, F. C. and Wisser, H. *Journal of Clinical Chemistry and Clinical Biochemistry*, (1978), **16**, 239.
[18] Chasson, A., Grady, H. T., Stanley, M. A. *American Journal of Clinical Pathology*, (1961), **35**, 83.
[19] Gustaffsson, J. E. C., *Clinical Chemistry*, (1976), **22**, 616.
[20] Henry, P., and Saunders, R. A. *Annals of Clinical Biochemistry*, (1975), **12**, 119.
[21] Barnett, R. N. *The American Journal of Clinical Pathology*, (1968), **50**, 671.

### Computer evaluation of the EMIT assays carbamazepine, ethosuximide, phenobarbital, phenytoin, quinidine and theophylline on the Gemsaec centrifugal fast analyser

**Bengt Kinberger and Bengt-Åke Johansson**
*Department of Clinical Chemistry, Central Hospital, 301 85 Halmstad, Sweden*

### Introduction

The adaptation of EMIT antiepileptic drug assays to the Aminco Roto Chem II centrigufal fast analyser has been described by Finley et al [1,2]. The analysis procedure was very convenient and accurate; however, most of the data handling had to be done with the aid of a HP 9815A desktop computer. Using Finley's modification of the EMIT assays the authors wished to adapt EMIT similarly to the Gemsaec centrifugal fast analyser and perform all the data processing with the Gemsaec computer. As no EMIT-program was available it was decided to develop one that would operate for routine clinical chemistry work. The program was written in FOCAL 8 (the version used by Electro Nucleonics Inc., the manufacturer of the Gemsaec) computer language. The Gemsaec transfer disc has only 16 positions, so it was considered important not to occupy a large part of the disc with standards when analysing unknown samples.

### Apparatus

A Gemsaec centrifugal fast analyser attached to a PDP 8/e computer, with magnetic tape (dectape) as the storage device, was used for the evaluation. The Rotor temperature was kept within 37.0 ± 0.1°C. Electro Nucleonics Inc.'s loader for the Gemini analyser was used for the preparation of transfer discs for Gemsaec. The loader was prepared for the automatic delivering of two reagents and the sample flushed with buffer solution into the transfer disc.

### Reagents

Reagent kits from the Syva Corporation were used through out the evaluation.

*Stock solutions*: Reagents A, B, aed-buffer and calibration standards were reconstituted according to Syva's recommendations.

